# Mind the gap! Extraluminal percutaneous-endoscopic rendezvous with a self-expanding metal stent for restoring continuity in major bile duct injury: A case series

**DOI:** 10.1016/j.ijscr.2019.06.059

**Published:** 2019-06-28

**Authors:** Jessica Lindemann, Christo Kloppers, Sean Burmeister, Marc Bernon, Eduard Jonas

**Affiliations:** a Surgical Gastroenterology Unit, Division of General Surgery, University of Cape Town Health Sciences Faculty and Groote Schuur Hospital, Cape Town, South Africa; b Department of Surgery, Washington University School of Medicine, Saint Louis, MO, USA

**Keywords:** Bile duct injury, Endoscopic percutaneous rendezvous, Self expanding metal stent, Minimally invasive

## Abstract

**Introduction:**

Treatment of major iatrogenic and non-iatrogenic bile duct injury (BDI) often requires delayed surgery with interim external biliary drainage. Percutaneous transhepatic cholangiography (PTC) with biliary catheter placement and endoscopic retrograde cholangiography (ERC) with stent placement have been used to bridge defects. In some patients, bridging the defect cannot be achieved through ERC or PTC alone.

**Materials and methods:**

Two patients with major BDIs, one iatrogenic and one non-iatrogenic underwent an extraluminal PTC/ERC rendezvous with placement of a fully covered self-expandable metal stent (SEMS) for the acute management of BDI with substantial loss of bile duct length.

**Results:**

In both patients the intraperitoneal PTC/ERC rendezvous with SEMS placement was successful with no complications after 12 and 18 months follow-up, respectively.

**Discussion:**

This study is the first to report a standardized approach to the acute management of iatrogenic and non-iatrogenic major BDIs using extraluminal intraperitoneal PTC/ERC rendezvous with placement of a fully covered SEMS. The described technique may serve as a “bridge to surgery” strategy for patients where definitive management of BDIs are deferred. However, long-term data of the success of this technique, specifically the use of a SEMS to bridge the defect, are lacking and further investigation is required to determine its role as a definitive treatment of BDIs with substance loss.

**Conclusion:**

PTC/ERC rendezvous with restoration of biliary continuity and internalization of bile flow is particularly useful for patients who have previously failed ERC and/or PTC alone, and in whom immediate surgical repair is not an option.

## Highlights

• Bile duct injuries with substance loss can be challenging to treat surgically.
• Morbid obesity, intra-abdominal sepsis and penetrating trauma increase complexity.
• Endoscopic/percutaneous techniques are alternative treatment options.
• Combined rendezvous with immediate stenting was successful in two challenging cases.

## 1 Introduction

The majority of BDIs occur as iatrogenic injury during cholecystectomy. Non-iatrogenic BDI after penetrating or blunt abdominal trauma is rare, reported in 0.1% of trauma admissions [1]. The standard approach to BDI involving disruption or complete transection of the common bile or hepatic ducts is hepaticojejunostomy [2]. However, surgical repair may result in significant morbidity and mortality [3]. In patients with iatrogenic BDI, reconstruction may be deferred for uncontrolled sepsis and to optimize the patient’s condition [4]. In this interval, the biliary fistula is controlled with percutaneous drains resulting in external loss of bile production, and possible fluid and electrolyte imbalances [5]. Maintaining or improving nutritional status during this period can be difficult due to the luminal absence of bile [6]. Over 90% of patients with non-iatrogenic BDI have other associated intra-abdominal injuries [1]. In a substantial percentage of these patients, initial surgery will be according to damage control principles. In this setting, the initial management of the BDI is similar to iatrogenic BDI with external drainage of bile and definitive repair at a later stage.

Several case reports and small case series have suggested a minimally invasive approach using percutaneous transhepatic and/or endoscopic access for establishing biliary continuity [[7], [8], [9]].

The use of a single modality to internalize bile drainage requires bridging the defect with a guidewire enabling placement of a PTBC or transpapillary stent. Rendezvous PTC/ERC has been described in patients where transpapillary cannulation of the bile duct is unsuccessful using ERC alone [10]. A guide-wire is passed transhepatic with the rendezvous occurring in the duodenum once the guidewire passes the papilla allowing ERC interventions [11]. We describe an extraluminal PTC/ERC rendezvous technique with placement of a fully covered SEMS for the acute management BDIs with substantial substance loss. The following presentation of two patient cases has been reported according to the PROCESS guidelines [12].

## 2 Materials and methods

Two patients with BDIs and substantial tissue loss, one iatrogenic and one non-iatrogenic, were included. Patient data were retrospectively retrieved from a prospective ERCP registry consisting of patients treated at a public academic hospital in Cape Town, South Africa. Additional information was collected from patient records. The demographic and clinical characteristics of the patients are presented in Table 1 and described below. In accordance with the declaration of Helsinki this case series was retrospectively registered in a publicly accessible database (UIN: 4868). Institutional ethics approval was obtained (HREC: 414/2018).

**Table 1:** Demographics and clinical information for two patients with bile duct injury and substantial loss of substance who were successfully treated with extraluminal PTC/ERC rendezvous.

Pre-rendezvous imaging	Patient 1 Iatrogenic BDI	Patient 2 Non-iatrogenic BDI
CT	Free fluid in abdomen	AAST Grade IV liver injury, subhepatic collection
MRI/MRCP	Complete transection of CHD	Complete transection of CHD
ERC	Complete transection of CBD	Complete transection of CBD
PTC drainage	Via segment 8 duct, 8 Fr pigtail	Via segment 6 duct, 8 Fr pigtail
**Characteristics of injury**		
Distance of injury from hepatic confluence (mm)	10 mm	10 mm
Length of substance loss (mm)	10 mm	25 mm
Diameter of associated collection (mm)	95 × 90 mm	72 × 45 mm

AAST – American association for the surgery of trauma [13], CHD – common hepatic duct, CBD – common bile duct, Fr – French.

### 2.1 Interventional technique

This intervention was performed by the same senior interventional radiologist and experienced endoscopists. After establishing the extent of the injury on cross-sectional imaging, a PTC is performed to confirm the anatomy, specifically assessing whether the biliary confluence is intact and if the length of remaining proximal CHD will allow placement of a fully covered SEMS. At PTC, a PTBC is passed through the severed bile duct into the subhepatic space for drainage of collections. Step 1 of the rendezvous intervention is performing an ERC with a distal cholangiogram (Endoscope, Olympus Exera III). Matching the PTC and ERC imaging, the extent of substance loss is determined. In Step 2, a stone retrieval basket is passed endoscopically through the distal end of the bile duct and opened in the subhepatic space. In Step 3, a standard 420 cm ERCP guidewire is passed transhepatic via the PTBC into the subhepatic space. In Step 4, the wire is caught in the basket under fluoroscopic guidance and pulled through the working channel of the duodenoscope out of the oral cavity after which the PTBC is removed. In Step 5, a fully covered SEMS (10 cm × 8 Fr; Boston Scientific, Boston MA) is deployed endoscopically, bridging the defect. Care is taken to have the proximal stent border distal to the biliary confluence, ensuring bilateral biliary drainage. In Step 6, using the guidewire already in place, a new antegrade placed PTBC is deployed through the stent to prevent bile leak from the puncture on the liver surface, maintain percutaneous access for further intervention if needed and catch a migrating stent, should it occur.

### 2.2 Patient 1

**Fig. 1:**
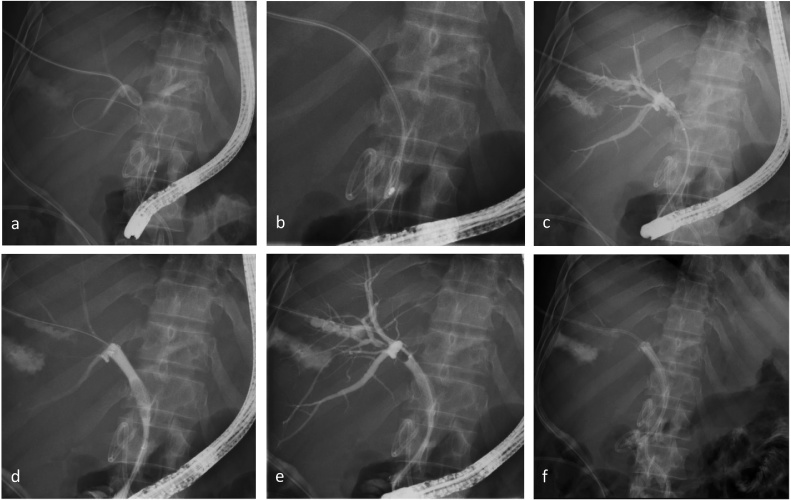
**Extraluminal endoscopic PTC/ERC rendezvous with simultaneous SEMS placement for management of an iatrogenic BDI**. Image 1a shows the PTBC in place prior to performing the rendezvous intervention. Image 1b demonstrates the initial cholangiogram at ERC (Step 1) as the endoscopist prepares to position and deploy the stone extraction basket in the subhepatic space (Step 2). In 1c the guidewire has been passed transhepatic through the PTBC access (Step 3) and caught using the stone retrieval basket (Step 4). Image 1d shows the fully covered SEMS not yet expanded in place, bridging the gap (Step 5). Image 1e shows the fully expanded stent with the transhepatic wire in place as a new PTBC is deployed (Step 6). Image 1f demonstrates the final result of the PTC/ERC rendezvous with right-sided PTBC internalized through a SEMS. PTC – percutaneous transhepatic cholangiogram, ERC – endoscopic retrograde cholangiography, SEMS – self-expanding metal stent, BDI – bile duct injury, PTBC – percutaneous transhepatic biliary catheter.

A 33-year-old morbidly obese female underwent an elective LC and was diagnosed with an iatrogenic BDI on post-operative day 22. She was taken for an exploratory laparotomy with washout and drainage and referred to our unit for further management five days later. Cross-sectional imaging confirmed a complete transection of the extrahepatic bile duct with 10 mm loss of substance. Due to uncontrolled sepsis the decision was made to defer definitive treatment. An ERC was performed that showed extravasation of contrast into the subhepatic space and no filling of the proximal bile ducts. After placement of a transhepatic drain an extraluminal rendezvous procedure was performed and a 10 × 80 mm SEMS placed, bridging the defect (Fig. 1).

### 2.3 Patient 2

**Fig. 2:**
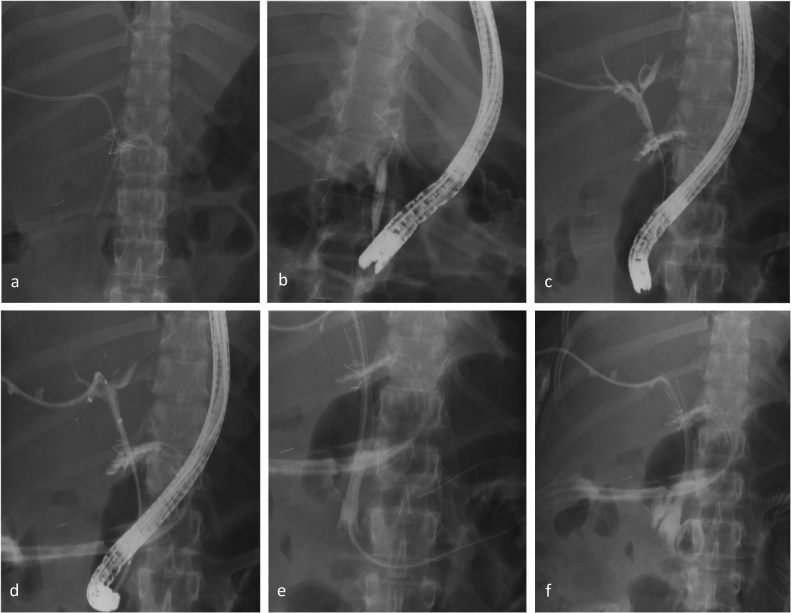
**Extraluminal endoscopic PTC/ERC rendezvous with simultaneous SEMS placement for management of a non-iatrogenic BDI**. Image 2a shows the ERC placed guidewire just before advancing the stone extraction basket into the subhepatic space (Step 2). Image 2b demonstrates the percutaneously placed guidewire caught in the stone extraction basket (Step 4). Image 2c illustrates a right sided cholangiogram performed prior to removal of the PTBC. Images 2d and 2e show deployment of the SEMS, bridging the gap (Step 5). Image 2f shows the final result of the PTC/ERC rendezvous with a right-sided PTBC internalized through the SEMS (Step 6). PTC – percutaneous transhepatic cholangiography, ERC – endoscopic retrograde cholangiography, SEMS – self-expanding mental stent, BDI – bile duct injury, PTBC – percutaneous transhepatic biliary catheter.

A 40-year-old male presented with a trans-axial thoraco-abdominal gunshot wound. He was hemodynamically unstable, and a damage control laparotomy was performed. Gastric and diaphragmatic injuries were repaired and a grade IV liver injury was packed [13]. The packs were removed after 24 h and a closed suction drain was left in the subhepatic space. Six days after laparotomy, CT abdomen showed non-perfusion of liver segments 2 and 3, a large central intrahepatic hematoma and a subhepatic collection. A percutaneous ultrasound-guided puncture of the collection returned bile and an 8 Fr pigtail drain was placed. He subsequently developed a persistent bile leak and rising serum bilirubin (13–38 mmol/L). ERC demonstrated extravasation of contrast into the subhepatic space and no filling of the proximal bile ducts. MRCP showed complete disruption of the extrahepatic bile duct but an intact confluence. A PTC was performed noting a porto-biliary fistula and an 8 Fr PTBC was positioned into the subhepatic space. At extraluminal PTC/ERC rendezvous a 10 × 80 mm fully covered SEMS was placed, bridging the defect (Fig. 2). The patient developed haemobilia 48 h later. Angiography showed a bleeding right hepatic artery false aneurism successfully managed with an endovascular stent.

## 3 Results

Patient 1 had no post-procedural complications and following embolization, Patient 2 made an uneventful recovery requiring no additional intervention. Pre- and post-rendezvous blood tests are shown in Table 2. Notably, albumin normalized within 3 months in both patients. The PTBC for Patient 1 was removed after 4 months and after 3 months for Patient 2. Both continue to be followed with regular outpatient visits and liver function tests. They remain asymptomatic after 12 and 18 months of follow-up respectively, with no long-term complications. Although the initial intention was internalization of bile flow while surgical repair was delayed, we have subsequently embarked on a definitive endoscopic strategy with stent changes every 3 months. In the absence of any previous experience or guidelines for this situation, an arbitrary total stent time of 24 months was chosen.

**Table 2:** Pre- and post-interventional blood tests of two patients with bile duct injury and substantial loss of substance who were successfully treated with extraluminal PTC/ERC rendezvous.

	Patient 1 Iatrogenic BDI		Patient 2 Non-iatrogenic BDI	
	Pre-intervention	3 months post-intervention	Pre-intervention	3 months post-intervention
TB (umol/L)	19	8	16	4
ALT (IU/L)	16	14	87	13
AST (IU/L)	23	16	52	16
GGT (IU/L)	69	33	415	26
ALP (IU/L)	166	86	135	90
Albumin (g/L)	24	39	27	43
Sodium (mmol/L)	137	138	133	145
Potassium (mmol/L)	3.9	4.3	5.2	4.2
Urea (mmol/L)	1.8	3.1	3.2	2.2
Creatinine (umol/L)	46	38	58	68
WCC (×10^9^/L)	12.50	7.17	15.68	6.64
Haemoglobin (g/L)	84	123	89	122
Platelets (×10^9^/L)	539	344	489	391
INR	1.56	1.27	–	1.03

TB – total bilirubin, ALT – alanine transaminase, AST – aspartate transaminase, GGT – gamma glutamyl transferase, ALP – alkaline phosphatase, WCC – white cell count, INR – international normalized ratio.

## 4 Discussion

The described technique may serve as a “bridge to surgery” strategy for patients where definitive management of BDIs are deferred. PTC/ERC rendezvous is particularly useful for patients who have previously failed ERC and/or PTC alone and in whom immediate surgical repair is not an option. An important advantage of internalization in this approach is that it prevents external bile fluid loss, optimizing nutritional status, and preventing electrolyte abnormalities and dehydration [5].

As forthcoming in the trauma patient, although rare, extrahepatic BDIs in trauma can present a formidable challenge due to the high incidence of associated injuries, especially vascular injuries. Often, definitive surgery is delayed for several weeks to months and even after delay can be technically difficult. In this group of patients, the definitive PTC/ERC rendezvous approach has the potential to minimize short- and long-term complications associated with non-iatrogenic BDIs.

The technique of extraluminal rendezvous has been described previously. Odemis et al. performed an intraperitoneal rendezvous procedure with placement of a single plastic stent into the right biliary system at the time of rendezvous in a patient with a complex BDI [14]. Over the next year, multiple additional plastic stents were place with resolution of the stricture. However, evidence of long-term success using a definitive stent strategy in these patients is lacking, especially long-term results for SEMS. In a series of 22 patients with complete bile duct transection after LC, 18 patients were asymptomatic and 4 underwent surgical repair after a mean follow-up period of 5 years [15]. Schreduer et al. found a long-term success rate of 55% in 47 patients after a median follow-up of 40 months [5]. Of note, only 31 of 47 patients had a complete transection of the bile duct and both studies report only patients with iatrogenic BDIs. Additionally, in both studies plastic stents were used. The use of SEMS for major BDIs in the acute setting has to our knowledge not been described previously. Although long-term results need to be confirmed the creation of a lumen substantially larger than plastic stents may contribute to better long-term results in these patients.

## 5 Conclusion

This is the first study to report the use of intraperitoneal PTC/ERC rendezvous with placement of a fully-covered SEMS to immediately bridge the gap in BDI, allowing internal biliary drainage in the acute management of patients with BDI and substance loss.

## Conflicts of interest

The authors have no financial or personal relationships resulting in conflicts of interest to disclose.

## Funding

There was no funding provided to complete this research.

## Ethical approval

This study was approved by the Human Research Ethics Committee at the University of Cape Town (ref 414/2018).

## Consent

The Human Research Ethics Committee (HREC) requires written consent to be obtained from all patients included in institutional databases, from which data for this report was retrieved. Written informed consent was obtained from the patients specifically for publication of this case report, including imaging used in the case presentations. A copy of the written consent is available for review by the Editor-in-Chief of this journal on request.

## Author’s contribution

Jessica Lindemann: Data collection and analysis, writing the paper, critical revisions, final approval.

Christo Kloppers: Study concept, data collection, critical revisions, final approval.

Sean Burmeister: Study concept, critical revisions, final approval.

Marc Bernon: Study concept, data collection, critical revisions, final approval.

Eduard Jonas: Study concept, data analysis, writing the paper, critical revisions, final approval.

## Registration of research studies

This research was retrospectively registered at researchregistry.com with the UIN: 4868.

## Guarantor

Professor Eduard Jonas.

## Provenance and peer review

Not commissioned, externally peer-reviewed.
